# Samples in randomized clinical trials with interim analysis

**DOI:** 10.17843/rpmesp.2023.402.12217

**Published:** 2023-06-30

**Authors:** Michelle Saaibi Meléndez, Felipe Botero-Rodríguez, Carlos Javier Rincón Rodríguez

**Affiliations:** 1 Semillero de Bioestadística, School of medicine, Pontificia Universidad Javeriana, Bogotá, Colombia. Pontificia Universidad Javeriana Semillero de Bioestadística School of medicine Pontificia Universidad Javeriana Bogotá Colombia; 2 Department of Clinical Epidemiology and Biostatistics, Pontificia Universidad Javeriana, Bogotá, Colombia. Pontificia Universidad Javeriana Department of Clinical Epidemiology and Biostatistics Pontificia Universidad Javeriana Bogotá Colombia

**Keywords:** Sample Size, Clinical Trials, Hypothesis Tests

## Abstract

This article introduces randomized clinical trials and basic concepts of statistical inference. We present methods for calculating the sample size by outcome type and the hypothesis to be tested, together with the code in the R programming language. We describe four methods for adjusting the original sample size for interim analyses. We sought to introduce these topics in a simple and concrete way, considering the mathematical expressions that support the results and their implementation in available statistical programs; therefore, bringing health students closer to statistics and the use of statistical programs, which are aspects that are rarely considered during their training.

## INTRODUCTION

The approach to medicine has shifted from an initial paternalistic view to pragmatic reductionism. This change occurred because of the drive to improve the quality of care, decrease individual economic incentives and prioritize the importance of research to improve the quality of evidence ^1^. Evidence-based medicine emerged as a new paradigm in the 1990s as scientific support for clinical decision-making and is based on a hierarchy of three statements: a) randomized clinical trials (RCTs) or systematic reviews of many experiments usually provide more evidence than observational studies; b) analytical clinical studies provide better evidence than pathophysiological rationale alone; and c) analytical clinical studies provide more evidence than expert judgment ^2^.

Obtaining valid results from RCTs depends on the quality of the data, which must be sufficient to address the research question. To obtain these quality data, the sample size must be large enough to obtain an accurate estimate of the effect of the intervention. Random errors will not affect the interpretability of the results as long as the sample is large enough; however, a systematic error can invalidate a study ^3^.

The interim analysis consists of setting an observation point(s), so the behavior of the sample can be assessed up until that point. Depending on the results, the committee may determine if the study is relevant enough to continue or not ^3^. This article seeks to provide an introduction to the calculation of sample size by type of outcome and hypothesis. We also aim to provide information on its adjustment by interim analysis, considering the mathematical formulas and their implementation in available statistical programs such as the R programming language. The objective is to bring health personnel closer to statistics and the use of programs, aspects that are little considered in their training. Although there are already several sources that develop the above topics, there are not many documents that merge both theory and practice, including all the aspects mentioned above regarding RCTs. Reviewing articles allows young researchers and health professionals to make a first approach to these topics, without generating an initial rejection due to their complexity. Connecting mathematical expressions with their implementation in a statistical program seeks to avoid that, once again, young researchers execute pre-established functions such as TwoSampleMean.Equality or TwoSampleMean.NIS (included in the “TrialSize” package ^4^) without understanding where the results come from, the effect of the parameters on the sample size or the need to choose parameters with values consistent with the type of hypothesis that is being evaluated. This seeks to promote understanding of the mechanical execution of tasks only to meet the requirements of an evaluation committee.

### Randomized clinical trials

The equipoise principle corresponds to a state of uncertainty regarding the therapeutic results of a treatment, which justifies an RCT ^5^. RCTs with a control group are prospective studies that compare the outcomes of an intervention(s) with the best available alternative. In these studies, patient safety should always be a priority, so the possible benefits, harms and treatment alternatives for the patient’s condition should be explained. Although it may have limitations, it is considered the best alternative for evaluating the efficacy or safety of an intervention ^6^^,^^7^. It is characterized by: a) an intervention that is compared with a control group that can be placebo or the usual treatment, b) randomized assignment of the interventions in the population to reduce possible confusion bias by obtaining homogeneous groups and the possibility of selection bias by avoiding foreseeing the group to which the patient is assigned c) the blinding of the treatment groups can be performed both for researchers, patients or analysts, which minimizes possible information biases ^6^^,^^7^.

The RCTs are divided into four phases. Phase I seeks to determine possible toxic effects, absorption, distribution and metabolism of the drug in a group of 20-80 healthy people. Phase II is conducted in a diseased population to determine the safety and efficacy of the drug, based on biological markers and evaluating adverse reactions. Phase III is performed when there is evidence on the safety and efficacy of the intervention and additional information is sought on the safety and effectiveness of the drug in a larger number of participants. The intervention is compared with the usual therapy or placebo in a long-term follow-up in order to identify possible side effects. In phase IV, after the molecule has been approved for marketing, it is compared with other existing products in the general population; pharmacovigilance is also carried out in order to look for adverse events not identified in phase III due to their low incidence or long periods of occurrence ^3^^,^^6^.

In this article we will focus on phase III and IV studies, which require a sample size calculation. Additionally, we will work on parallel RCTs characterized by a simultaneous follow-up of each group to which they were assigned ^3^.

### Inferential statistics

Inferential statistics allows estimating the behavior of the entire population from the results obtained in a sample. This behavior is summarized in measures such as means, proportions or variances, which, if obtained for the whole population, would be called parameters ^7^. There are two alternatives: confidence intervals and hypothesis tests; with consistent results, the first seeks a range of values that, with a degree of confidence, contains the parameter of interest, while the second evaluates a statement about the parameter of interest, making the decision to reject it or not.

Since this paper presents the sample size calculation in parallel RCTs to evaluate parameter statements, we will describe the process of hypothesis testing. Initially, two hypotheses are proposed, the null hypothesis (*H*_*0*_ ) which is a statement about the parameter, and the alternative hypothesis (*H*_*a*_ ) which is its negation; almost always the alternative hypothesis ^8^, which is related to the research question is sought to be tested; at the end a decision is made to reject or not the *H*_*0*_ . Taking into account that this decision depends on the results obtained only from a sample, there is the probability of committing two errors, type I error or significance level (*α*) that occurs when rejecting *H*_*0*_ when it is true, and type II error (β), occurs when not rejecting *H*_*0*_ when it is false ^9^. The opposite of type I error is the confidence level (1-α) and corresponds to the probability of not rejecting H_0_ when it is true, and the opposite of type II error (1-β), which is the power, is the probability of rejecting *H*_*0*_ when it is false ^9^. When performing a hypothesis test, the probability of committing the type I and II error is low, which implies that the confidence level and power have a high probability (typically: α=0.05 and β=0.1 or 0.2). In order to guarantee these values, it is necessary to calculate the sample size.

In order to make the decision to reject *H*_*0*_ , an operation is carried with the values from the sample (test statistic) and contrasted with the behavior that should occur if *H*_*0*_ were true. If the value found by the test statistic is unlikely, this is evidence that *H*_*0*_ is false and is rejected in favor of *H*_*a*_ , otherwise it is considered that there is not enough evidence to reject *H*_*0*_ . The probability reflecting the evidence “for” or “against” *H*_*0*_ is called the p-value ^10^^,^^11^, and is equal to the probability, assuming the null hypothesis is true, of obtaining a value of the test statistic “...as extreme or more (in the appropriate direction of *H*_*a*_ ) than the value actually calculated” ^11^^,^^12^; finally, *H*_*0*_ is rejected under the assumption of a value of p<α. Statistical significance, commonly evaluated by means of the p-value, does not account for clinical significance; we speak of statistical significance when the premise of a value of p<α is fulfilled, while clinical significance is defined by those results that improve the physical, mental and social functionality of the patient, which can lead to an improvement in the quality or quantity of life, depending on the context ^14^.

### Types of hypotheses

There can be different research questions in an RCT that relate to four different ways of stating the *H*_*0*_ . The sample size calculation depends on the type of hypothesis to be tested; therefore, Table 1 presents their definitions along with an example.

**Table 1:** Types of hypotheses in randomized clinical trials.

Type	Definition ^24^	Hypothesis ^25^^,^^26^	Example	
			Hypothesis	Interpretation
Equality	Evaluates whether there are differences between the treatment and control groups.	H_0_: There is no difference between the two therapies.	Pressure over the estimated sternal projection of the aortic valve at the sternum is not associated with a change in hemodynamic parameters in the hypotensive patient.	Patients who underwent a pressure of 6 mm depth over the estimated sternal projection of the aortic valve on the sternum, maintained for 90 seconds, showed a homogeneous decrease of blood pressure and heart rate parameters ^27^.
		H_a_: There is a difference between the two therapies.	Pressure over the estimated sternal projection of the aortic valve on the sternum is associated with a change in hemodynamic parameters in the hypotensive patient.	
Non-inferiority	It evaluates whether the effect of a new treatment (whose effect is lower than the conventional treatment, but greater than the placebo) is within an accepted range and is established on the basis of the best available evidence. This difference is justified by side effects or feasibility.	H_0_: The effect of the new intervention is less than or equal to the placebo.	The new antimicrobial has the same effectiveness as the placebo.	The new antimicrobial, although better tolerated than conventional therapy, is less effective clinically and statistically, so it cannot be recommended as first line _(28)_.
		H_a_: The effect of the new intervention is greater than the placebo.	The new antimicrobial is more effective than the placebo.	
Superiority	Seeks to evaluate whether a new intervention generates better clinical outcomes than a well-established therapy or placebo.	H_0_: The new intervention is not superior to the established therapy.	Volunteering does not reduce social isolation or impact better mental health outcomes.	Volunteering did not prove to be superior compared to the control group regarding mental health outcomes or isolation ^29^.
		H_a_: New intervention is superior to established therapy.	Volunteering reduces social isolation and impacts better mental health outcomes.	
Equivalence	It seeks to evaluate whether the effect of the treatment is identical to that of another therapy.	H_0_: Therapies are not equivalent.	The inclusion of metformin, associated with oral contraceptives in the treatment of polycystic ovary syndrome, is not as effective as monotherapy with oral contraceptives alone.	The ultrasound remission time was shorter, there were less symptoms and the recurrence rate at 3 months was lower with the combined therapy, which shows greater effectiveness compared to the study group that received monotherapy ^30^.
		H_a_: The therapies are equivalent.	Oral contraceptive monotherapy is as effective as oral contraceptive therapy plus metformin for the treatment of polycystic ovary syndrome.	

H_0_: null hypothesis, H_a_: alternative hypothesis.

### Sample size

Generally, it is not possible to study the entire population, therefore, a specific sample size (n) is required to represent its behavior. As the sample size increases, the results approach that of the population, so that from a specific size, the results will not present large changes, making it unnecessary to continue collecting participants ^15^. Recruiting more subjects than necessary increases both the complexity of the logistical operation and the costs, and poses an ethical dilemma by unnecessarily assigning subjects to a treatment that has not proven its benefit. On the other hand, defining a very small sample size implies a high risk of the type II error mentioned above. The calculation of the sample size makes it possible to determine whether a study is feasible based on *a priori* assumptions, given the power, significance and background of previous studies addressing the same research question, taking into account the ethical considerations of subjecting people to an experiment ^13^^,^^16^.

In addition, when conducting an RCT, the possibility arises of observing the results obtained as the sample is collected. This is called “interim analyses”, which should be planned from the beginning of the investigation during the preparation of the protocol. These additional analyses increase the possibility of type I and II errors, and for this reason, the sample size must be adjusted to maintain a level of confidence and overall power throughout the RCT. The above reflects the importance of the sample size calculation, therefore, this article presents how to calculate the sample size for RCTs, showing the expression from which it is obtained, and its application using the R programming language ^17^. Additionally, we present how to perform the adjustment for interim analysis together with an example.

## MATERIALS AND METHODS

Based on the review of the book “Sample size calculations in clinical research” by Chow *et al*. ^13^, this article presents how to calculate the sample size for a parallel RCT, by: 1) type of outcome (dichotomous, continuous) and 2) type of hypothesis to be evaluated (equality, non-inferiority, superiority and equivalence). The corresponding mathematical expressions and the code to create a function in the R ^17^ and RStudio ^18^ programs are included. For the use of this code, the reader is required to have a basic knowledge of the use of these programs, where the function must be copied and executed; the function can then be used including the required parameters described in the results section. For each scenario, an example with fictitious data is included, specific considerations related to the function parameters are mentioned as well.

The methods of Pocock, O’Brien and Fleming, Wang and Tsiatis and Inner Wedge to adjust the original sample size obtained from the functions created previously in order to perform the interim analysis are described below. The adjustment consists of multiplying the original sample size by the coefficients included in Annexes 1 to 4 depending on the method used, and considering the number of planned evaluations (R), the power and significance level defined for the study. In addition, the expression of the test statistic used for each evaluation by type of outcome is included, based on the information of the participants entering the study. In summary, we present the following for each method: 1) the critical values that correspond to the values of the standard normal distribution that determine the rejection zone for evaluating the null hypothesis at each point in time, and 2) the coefficients for adjusting the sample size calculation.

### Sample size calculation for a dichotomous outcome

As an example, we assume that two treatments are to be compared and the outcome of interest is the proportion of deaths. For all expressions below, we denote *p*_*T*_ and *p*_*C*_ as the proportion of deceased in the treatment and control group, respectively; ϵ is the expected difference between these two proportions *(ϵ=p*_*T*_ *-p*_*C*_ *)*, *δ* is the margin of tolerance or superiority defined by the researchers, and k is the ratio between the sample size of the treatment group and the control group *(k=n*_*T*_ */n*_*C*_ *)*, i.e., *n*_*T*_ *=kn*_*C*_ . Finally, we denote *α* and *β* as the type I and II error, respectively; and *z*_*(q)*_ as the q percentile of the standard normal distribution function. In Table 2, we present the expressions to obtain *n*_*C*_ , and, in the inset, the code in the R programming language that creates a function for its implementation, along with an example where *α=0.05*, *β =0.2* and *k=1*.

**Table 2:**
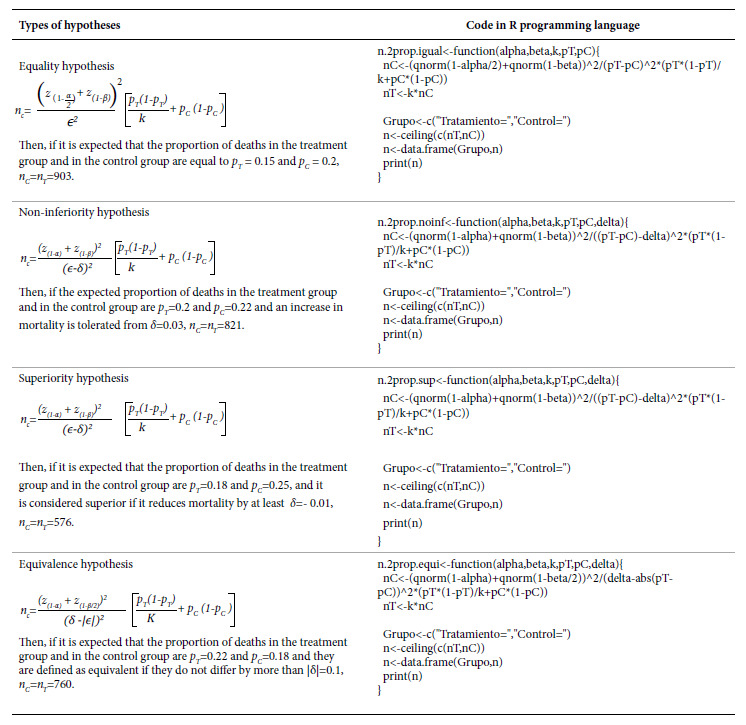
Types of hypotheses with dichotomous outcome and their code in the R programming language.

In all four hypotheses, the smaller the expected difference *(ϵ)* and the closer the proportions are to 0.5, the larger the sample size. When testing a non-inferiority hypothesis, if the higher the proportion of the event the greater the effectiveness, then *δ<0*; if the lower the proportion of the event the greater the effectiveness, then *δ>0*. When testing a superiority hypothesis, if the higher the proportion of the event the greater the effectiveness, then *δ>0*; if the lower the proportion of the outcome the greater the effectiveness, then *δ<0*. When testing an equivalence hypothesis, always *δ>0*.

### Continuous outcome sample size calculation

As an example, we assume that two treatments are to be compared, and the outcome is systolic blood pressure in mmHg (SBP). For all the expressions presented below, we denote *μ*_*T*_ and *μ*_*C*_ as the mean SBP in the treatment and control group, respectively; *ϵ* is the expected difference between the two means *ϵ=μ*_*T-*_ *μ*_*C*_ and is the standard deviation of the two samples together. *δ*, *k*, *α*, *β* and z_*(q)*_ represent the same values as in the previous section. In Table 3, we present the expressions to obtain the code in the R programming language for implementation with an example where *α=0.05*, *β=0.2* and *k=1*.

**Table 3:**
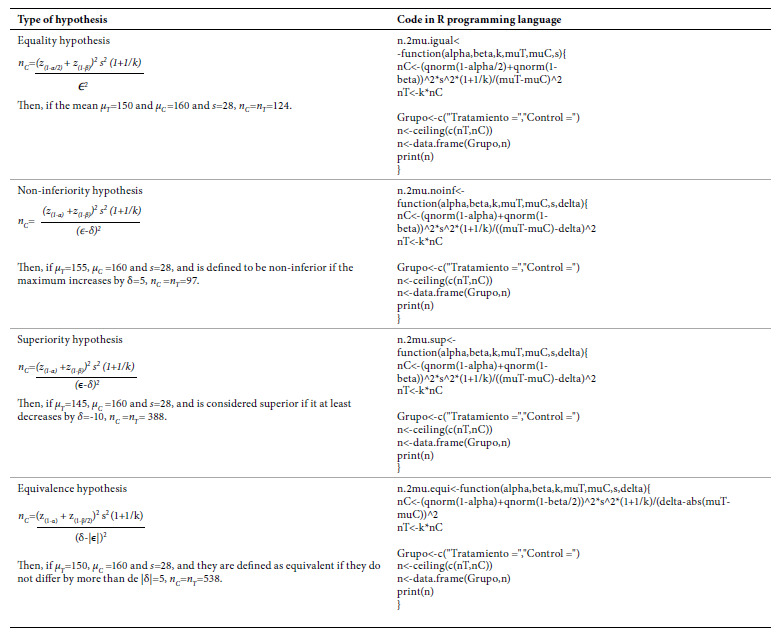
Types of hypotheses by continuous outcome and code in R programming language.

In all four hypotheses, higher *s* and lower *ϵ* require larger sample sizes. While testing a hypothesis of noninferiority, if the higher *μ* the greater the effectiveness, then *δ<0*; if the lower *μ* the greater the effectiveness, then *δ>0*. When testing a superiority hypothesis, if the higher *μ* the greater the effectiveness, then *δ>0*; if the lower *μ* the greater the effectiveness, then *δ<0*. When testing an equivalence hypothesis, always *δ>0*.

## RESULTS

### Interim Analysis

In an RCT, the study hypothesis can be tested sequentially as the sample is collected, giving the possibility of interrupting the collection if a clear benefit of the intervention is identified early. Depending on the number of evaluations *(R)* that are programmed, it is necessary to adjust the initial sample size to maintain the overall significance level of the study, and to establish the critical values on the distribution of the test statistic to reject or accept the null hypothesis in each evaluation. *R* evaluations are performed as n/R subjects accumulate, and the test statistic *z*_*r*_ *(r=1,2,...,R)* for a dichotomous outcome is equal to:


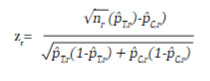


where *n*_*r*_ , *p̂*_*T,r*_ and *p̂*_*C,r*_ are the sample size per intervention group and the estimated proportions of the outcome at the *r* time of assessment of the treatment group and the control group, respectively.

For a continuous outcome, the test statistic is equal to:


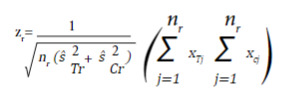


where n_r_, 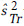 and 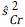 are the sample size in each intervention group, and the estimated variances at the time of the r_th evaluation of the treatment group and the control group, respectively. *x*_*Tj*_ and *x*_*Cj*_ are the observed values of the outcome in each subject collected until time r.

We present four methods that allow the adjustment of the sample size depending on the number of programmed evaluations, the significance level and the power established in a hypothesis of equality. First, the Pocock method, in which the sample size is adjusted by multiplying the sample size initially obtained from the expressions presented in the previous section, by the coefficients included in Annex 1, depending on the number of evaluations and the significance and power levels established. Now, if |*z*_*r*_ |>*CP*_*(r,α),*_ the *H*_*0*_ is rejected and data collection is suspended, otherwise, the collection continues. The critical values *CP*_*(r,α)*_ are presented in Annex 1 for defined *R* and *α*. The second method is that of O’Brien and Fleming and the coefficients to perform the initial sample size adjustment are presented in Annex 2. In this approach *H*_*0*_ is rejected at each evaluation if |*z*_*r*_ |>*COF*_*(r,α)*_ *√(R/r)*, otherwise it continues. The critical values *COF*_*(r,α)*_ are presented in Annex 2 according to the number of evaluations and significance level. The third method is that of Wang and Tsiatis, which includes a new parameter Δ ; coefficients for sample size adjustment for α=0.05 are included in Appendix 3. In this method *H*_*0*_ is rejected if 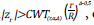 otherwise it continues. The critical *CWT*_*(r,α,Δ)*_ values are presented in Annex 3 for α=0.05. The methods of Pocock and O’Brien and Fleming are particular cases of the method of Wang and Tsiatis when Δ=0.5 and Δ=0, respectively, therefore, the critical values for these values of Δ are obtained from Annexes 1 and 2.

Finally, we present the Inner Wedge method; in this method unlike the previous three, two critical values are proposed: if |*zr*| ≥*b*_*r*_ rejects *H*_*0*_ and collection is suspended, the conclusion is that a significant treatment effect was found, on the other side, if |*zr*| <*a*_*r*_ does not reject *H*_*0*_ and collection is suspended the conclusion is that no differences between treatment and control are going to be found, otherwise, collection continues.

The critical values *a*_*r*_ and *b*_*r*_ are equal to:


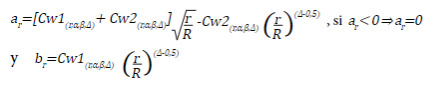


Annex 4 presents the values of *Cw*1 and *Cw*2 for an α=0.05 and a power of 0.8 and 0.9, and the columns *coef.fit* include the coefficients, by which the original sample must be multiplied to perform the R evaluations.

As an example, consider that you want to compare drug A vs. placebo and the outcome is the proportion of deaths at the end of follow-up. In a hypothesis of equality, assuming *α=0.05, β=0.1, p*_*T*_ *=0.1* and *p*_*C*_ *=0.2* (i.e. *ϵ*=0.1), for two groups of the same size, k=1, the required sample size in each group is 263 subjects. If we plan to perform R=5 evaluations, the adjusted sample size by Pocock’s method is 263 x 1.207=318 for each group and the critical value in each evaluation is *CP*_0.05,r_=2.413. The adjusted sample size by the method of O'Brien and Fleming is 263 x 1,026=270 for each group and the critical values for each evaluation are *COF*_*(r,0,05)*_ =4.562; 3.226; 2.634; 2.281 and 2.040. The adjusted sample size with the method of Wang and Tsiatis for each group, with Δ=0.25, would be 263 x 1.066=281, and the critical values at each evaluation would be *CWT*_*(r;0.05;0.25)*_ =3.194; 2.686; 2.427; 2.259 and 2.136. Finally, the adjusted sample size with the Inner Wedge method for each group would be, with Δ=0.25, 263 x 1.199=316, and the critical values for each evaluation are *a*_*r*_ =0;0.388; 1.072; 1.613 and 2.073 and *b*_*r*_ =3.1; 2.607; 2.355; 2.192 and 2.073.

## DISCUSSION

In this article we present an approach to sample size adjustment by interim analysis in parallel RCTs, starting from the calculation of the original sample size for subsequent adjustment by one of the four methods described. This paper is aimed at students and young researchers, mainly from the health area, who will find in this article an initial context on RCTs and a review of the main concepts of statistical inference from hypothesis testing. We seek, in a simple and concrete way, to provide an introduction to this topic, integrating the different aspects such as the mathematical expressions that support the results and their implementation in available statistical programs. Although there are other resources available for the calculation of sample size such as Internet pages ^19^ or packages in the R programming language ^4^, mainly in languages other than Spanish, we found that providing the possibility of using statistical programs that allow students to apply the theory gives a greater understanding of these topics, as opposed to following a sequence of steps in a mechanical way, often without understanding what is generated by the different programs or resources available. This brings students of health areas closer to statistics and the use of statistical programs, an aspect often considered not important during their training.

This article allows the reader to plan an RCT in parallel by defining the sample size and allowing the results to be monitored during the course of the study. At this point, we recommend reviewing additional methods that provide more flexibility, for example, planning the intermediate evaluations on specific dates and not when a fixed number of participants in both groups are completed, which is the main restriction for the four methods presented in this article. The method proposed by Lan and DeMets ^20^ and R programming language packages such as gsDesign ^21^ would be interesting material to further explore these issues.

Finally, the decision to discontinue the execution of an RCT, either because of great benefits, potential harms or if it is very unlikely to obtain benefits (futility), should be taken by a group of people independent of the researchers, made up of experts in the clinical area under study, in methodological aspects such as epidemiologists or biostatisticians, and in ethical aspects ^3^^,^^22^^,^^23^. All the necessary aspects should be considered in this decision and not only the result of the evaluation of a statistical test. Planning an interim analysis by adjusting the sample size when the study is being designed will allow supporting, to a greater extent, the value of this criterion during the decision-making process.
