# Supplementary material for: Samples in randomized clinical trials with interim analysis
Source: Rev Peru Med Exp Salud Publica. 2023 Jun 30;40(2):220–8. doi: 10.17843/rpmesp.2023.402.12217 (PMC10953660; doi:10.17843/rpmesp.2023.402.12217)
Supplement: Supplementary material. — Available in the electronic version of the RPMESP. [file rpmesp-40-02-12217-s001.docx]

**Anexos**

**Anexo 1.** Coeficiente de ajuste del tamaño de muestra y valores críticos por el método de *Pocock*.

| R | Coeficientes de ajuste | | | | | | Valores críticos | | |
| --- | --- | --- | --- | --- | --- | --- | --- | --- | --- |
|  | $\mathbf{1-}\boldsymbol{\beta}\mathbf{=0,8}$ | | | $\mathbf{1-}\boldsymbol{\beta}\mathbf{=0,9}$ | | |  |  |  |
|  | $\boldsymbol{\alpha}\mathbf{=0,01}$ | $\boldsymbol{\alpha}\mathbf{=0,05}$ | $\boldsymbol{\alpha}\mathbf{=0,1}$ | $\boldsymbol{\alpha}\mathbf{=0,01}$ | $\boldsymbol{\alpha}\mathbf{=0,05}$ | $\boldsymbol{\alpha}\mathbf{=0,1}$ | $\boldsymbol{\alpha}\mathbf{=0,01}$ | $\boldsymbol{\alpha}\mathbf{=0,05}$ | $\boldsymbol{\alpha}\mathbf{=0,1}$ |
| 1 | 1,000 | 1,000 | 1,000 | 1,000 | 1,000 | 1,000 | 2,576 | 1,960 | 1,645 |
| 2 | 1,092 | 1,110 | 1,121 | 1,084 | 1,100 | 1,110 | 2,772 | 2,178 | 1,875 |
| 3 | 1,137 | 1,166 | 1,184 | 1,125 | 1,151 | 1,166 | 2,873 | 2,289 | 1,992 |
| 4 | 1,166 | 1,202 | 1,224 | 1,152 | 1,183 | 1,202 | 2,939 | 2,361 | 2,067 |
| 5 | 1,187 | 1,229 | 1,254 | 1,170 | 1,207 | 1,228 | 2,986 | 2,413 | 2,122 |
| 6 | 1,203 | 1,249 | 1,277 | 1,185 | 1,225 | 1,249 | 3,023 | 2,453 | 2,164 |
| 7 | 1,216 | 1,265 | 1,296 | 1,197 | 1,239 | 1,266 | 3,053 | 2,485 | 2,197 |
| 8 | 1,226 | 1,279 | 1,311 | 1,206 | 1,252 | 1,280 | 3,078 | 2,512 | 2,225 |
| 9 | 1,236 | 1,291 | 1,325 | 1,215 | 1,262 | 1,292 | 3,099 | 2,535 | 2,249 |
| 10 | 1,243 | 1,301 | 1,337 | 1,222 | 1,271 | 1,302 | 3,117 | 2,555 | 2,270 |
| 11 | 1,250 | 1,310 | 1,348 | 1,228 | 1,279 | 1,312 | 3,133 | 2,572 | 2,288 |
| 12 | 1,257 | 1,318 | 1,357 | 1,234 | 1,287 | 1,320 | 3,147 | 2,588 | 2,304 |
| 15 | 1,272 | 1,338 | 1,381 | 1,248 | 1,305 | 1,341 | 3,182 | 2,626 | 2,344 |
| 20 | 1,291 | 1,363 | 1,411 | 1,264 | 1,327 | 1,367 | 3,225 | 2,672 | 2,392 |

**Anexo 2.** Coeficiente de ajuste del tamaño de muestra y valores críticos por el método de *O’Brien* y *Fleming*.

| **R** | **Coeficientes de ajuste** | | | | | | **Valores críticos** | | |
| --- | --- | --- | --- | --- | --- | --- | --- | --- | --- |
|  | $\mathbf{1-}\boldsymbol{\beta}\mathbf{=0,8}$ | | | $\mathbf{1-}\boldsymbol{\beta}\mathbf{=0,9}$ | | |  |  |  |
|  | $\boldsymbol{\alpha}\mathbf{=0,01}$ | $\boldsymbol{\alpha}\mathbf{=0,05}$ | $\boldsymbol{\alpha}\mathbf{=0,1}$ | $\boldsymbol{\alpha}\mathbf{=0,01}$ | $\boldsymbol{\alpha}\mathbf{=0,05}$ | $\boldsymbol{\alpha}\mathbf{=0,1}$ | $\boldsymbol{\alpha}\mathbf{=0,01}$ | $\boldsymbol{\alpha}\mathbf{=0,05}$ | $\boldsymbol{\alpha}\mathbf{=0,1}$ |
| 1 | 1,000 | 1,000 | 1,000 | 1,000 | 1,000 | 1,000 | 2,576 | 1,960 | 1,645 |
| 2 | 1,001 | 1,008 | 1,016 | 1,001 | 1,007 | 1,014 | 2,580 | 1,977 | 1,678 |
| 3 | 1,007 | 1,017 | 1,027 | 1,006 | 1,016 | 1,025 | 2,595 | 2,004 | 1,710 |
| 4 | 1,011 | 1,024 | 1,035 | 1,010 | 1,022 | 1,032 | 2,609 | 2,024 | 1,733 |
| 5 | 1,015 | 1,028 | 1,040 | 1,014 | 1,026 | 1,037 | 2,621 | 2,040 | 1,751 |
| 6 | 1,017 | 1,032 | 1,044 | 1,016 | 1,030 | 1,041 | 2,631 | 2,053 | 1,765 |
| 7 | 1,019 | 1,035 | 1,047 | 1,018 | 1,032 | 1,044 | 2,640 | 2,063 | 1,776 |
| 8 | 1,021 | 1,037 | 1,049 | 1,020 | 1,034 | 1,046 | 2,648 | 2,072 | 1,786 |
| 9 | 1,022 | 1,038 | 1,051 | 1,021 | 1,036 | 1,048 | 2,654 | 2,080 | 1,794 |
| 10 | 1,024 | 1,040 | 1,053 | 1,022 | 1,037 | 1,049 | 1,660 | 2,087 | 1,801 |
| 11 | 1,025 | 1,041 | 1,054 | 1,023 | 1,039 | 1,051 | 2,665 | 2,092 | 1,807 |
| 12 | 1,026 | 1,042 | 1,055 | 1,024 | 1,040 | 1,052 | 2,670 | 2,098 | 1,813 |
| 15 | 1,028 | 1,045 | 1,058 | 1,026 | 1,042 | 1,054 | 2,681 | 2,110 | 1,826 |
| 20 | 1,030 | 1,047 | 1,061 | 1,029 | 1,045 | 1,057 | 2,695 | 2,126 | 1,842 |

**Anexo 3**. Coeficientes para el ajuste del tamaño de muestra y valores críticos por el método de *Wang* y *Tsiatis*.

| **R** | **Coeficientes de ajuste** | | | | | | **Valores críticos (**$\boldsymbol{\alpha}\boldsymbol{=}\boldsymbol{0}\boldsymbol{,}\boldsymbol{05}$**)** | | |
| --- | --- | --- | --- | --- | --- | --- | --- | --- | --- |
|  | $\mathbf{1-}\boldsymbol{\beta}\mathbf{=0,8}$ | | | $\mathbf{1-}\boldsymbol{\beta}\mathbf{=0,9}$ | | |  |  |  |
|  | $\boldsymbol{\Delta}\mathbf{=0,1}$ | $\boldsymbol{\Delta}\mathbf{=0,25}$ | $\boldsymbol{\Delta}\mathbf{=0,4}$ | $\boldsymbol{\Delta}\mathbf{=0,1}$ | $\boldsymbol{\Delta}\mathbf{=0,25}$ | $\boldsymbol{\Delta}\mathbf{=0,4}$ | $\boldsymbol{\Delta}\mathbf{=0,1}$ | $\boldsymbol{\Delta}\mathbf{=0,25}$ | $\boldsymbol{\Delta}\mathbf{=0,4}$ |
| 1 | 1,000 | 1,000 | 1,000 | 1,000 | 1,000 | 1,000 | 1,960 | 1,960 | 1,960 |
| 2 | 1,016 | 1,038 | 1,075 | 1,014 | 1,034 | 1,068 | 1,994 | 2,038 | 2,111 |
| 3 | 1,027 | 1,054 | 1,108 | 1,025 | 1,050 | 1,099 | 2,026 | 2,083 | 2,186 |
| 4 | 1,035 | 1,065 | 1,128 | 1,032 | 1,059 | 1,117 | 2,050 | 2,113 | 2,233 |
| 5 | 1,040 | 1,072 | 1,142 | 1,037 | 1,066 | 1,129 | 2,068 | 2,136 | 2,267 |
| 6 | 1,044 | 1,077 | 1,152 | 1,041 | 1,071 | 1,138 | 2,083 | 2,154 | 2,292 |
| 7 | 1,047 | 1,081 | 1,159 | 1,044 | 1,075 | 1,145 | 2,094 | 2,168 | 2,313 |
| 8 | 1,050 | 1,084 | 1,165 | 1,046 | 1,078 | 1,151 | 2,104 | 2,180 | 2,329 |
| 9 | 1,052 | 1,087 | 1,170 | 1,048 | 1,081 | 1,155 | 2,113 | 2,190 | 2,343 |
| 10 | 1,054 | 1,089 | 1,175 | 1,050 | 1,083 | 1,159 | 2,120 | 2,199 | 2,355 |
| 11 | 1,055 | 1,091 | 1,178 | 1,051 | 1,085 | 1,163 | 2,126 | 2,206 | 2,366 |
| 12 | 1,056 | 1,093 | 1,181 | 1,053 | 1,086 | 1,166 | 2,132 | 2,213 | 2,375 |
| 15 | 1,059 | 1,097 | 1,189 | 1,055 | 1,090 | 1,172 | 2,146 | 2,229 | 2,397 |
| 20 | 1,062 | 1,101 | 1,197 | 1,058 | 1,094 | 1,180 | 2,162 | 2,248 | 2,423 |

**Anexo 4.** Coeficientes de ajuste del tamaño de muestra y valores críticos para el método *Inner Wedge*.

| $\boldsymbol{\Delta}$ | $\boldsymbol{R}$ | $\left( \boldsymbol{1-\beta} \right)\mathbf{=0,8}$ | | | $\boldsymbol{(1-\beta)=0,9}$ | | |
| --- | --- | --- | --- | --- | --- | --- | --- |
|  |  | $\boldsymbol{Cw}\mathbf{1}$ | $\boldsymbol{Cw}\mathbf{2}$ | $\boldsymbol{Coef}\mathbf{.}\boldsymbol{Ajuste}$ | $\boldsymbol{Cw}\mathbf{1}$ | $\boldsymbol{Cw}\mathbf{2}$ | $\boldsymbol{Coef}\mathbf{.}\boldsymbol{Ajuste}$ |
| −0,50 | 1 | 1,960 | 0,842 | 1,000 | 1,960 | 1,282 | 1,000 |
|  | 2 | 1,949 | 0,867 | 1,010 | 1,960 | 1,282 | 1,000 |
|  | 3 | 1,933 | 0,901 | 1,023 | 1,952 | 1,305 | 1,010 |
|  | 4 | 1,929 | 0,919 | 1,033 | 1,952 | 1,316 | 1,016 |
|  | 5 | 1,927 | 0,932 | 1,041 | 1,952 | 1,326 | 1,023 |
|  | 10 | 1,928 | 0,964 | 1,066 | 1,958 | 1,351 | 1,042 |
|  | 15 | 1,931 | 0,979 | 1,078 | 1,963 | 1,363 | 1,053 |
|  | 20 | 1,932 | 0,988 | 1,087 | 1,967 | 1,370 | 1,060 |
| -0,25 | 1 | 1,960 | 0,842 | 1,000 | 1,960 | 1,282 | 1,000 |
|  | 2 | 1,936 | 0,902 | 1,026 | 1,957 | 1,294 | 1,006 |
|  | 3 | 1,932 | 0,925 | 1,040 | 1,954 | 1,325 | 1,023 |
|  | 4 | 1,930 | 0,953 | 1,059 | 1,958 | 1,337 | 1,033 |
|  | 5 | 1,934 | 0,958 | 1,066 | 1,960 | 1,351 | 1,043 |
|  | 10 | 1,942 | 0,999 | 1,102 | 1,975 | 1,379 | 1,071 |
|  | 15 | 1,948 | 1,017 | 1,120 | 1,982 | 1,394 | 1,085 |
|  | 20 | 1,952 | 1,027 | 1,131 | 1,988 | 1,403 | 1,094 |
| 0,00 | 1 | 1,960 | 0,842 | 1,000 | 1,960 | 1,282 | 1,000 |
|  | 2 | 1,935 | 0,948 | 1,058 | 1,958 | 1,336 | 1,032 |
|  | 3 | 1,950 | 0,955 | 1,075 | 1,971 | 1,353 | 1,051 |
|  | 4 | 1,953 | 0,995 | 1,107 | 1,979 | 1,381 | 1,075 |
|  | 5 | 1,958 | 1,017 | 1,128 | 1,990 | 1,385 | 1,084 |
|  | 10 | 1,980 | 1,057 | 1,175 | 2,013 | 1,428 | 1,127 |
|  | 15 | 1,991 | 1,075 | 1,198 | 2,026 | 1447 | 1,148 |
|  | 20 | 1,998 | 1,087 | 1,212 | 2,034 | 1,458 | 1,160 |
| 0,25 | 1 | 1,960 | 0,842 | 1,000 | 1,960 | 1,282 | 1,000 |
|  | 2 | 1,982 | 1,000 | 1,133 | 2,003 | 1,398 | 1,100 |
|  | 3 | 2,009 | 1,059 | 1,199 | 2,037 | 1,422 | 1,139 |
|  | 4 | 2,034 | 1,059 | 1,219 | 2,058 | 1,443 | 1,167 |
|  | 5 | 2,048 | 1,088 | 1,252 | 2,073 | 1,477 | 1,199 |
|  | 10 | 2,088 | 1,156 | 1,341 | 2,119 | 1,521 | 1,261 |
|  | 15 | 2,109 | 1,180 | 1,379 | 2,140 | 1,551 | 1,297 |
|  | 20 | 2,122 | 1,195 | 1,40 | 2,154 | 1,565 | 1,316 |
