# Supplementary material for: Samples in randomized clinical trials with interim analysis
Source: Rev Peru Med Exp Salud Publica. 2023 Jun 30;40(2):220–8. doi: 10.17843/rpmesp.2023.402.12217 (PMC10953660; doi:10.17843/rpmesp.2023.402.12217)
Supplement: Translation

# Translation: La muestra en ensayos clínicos aleatorizados con análisis interino

**Authors:** Michelle Saaibi Meléndez, Felipe Botero-Rodríguez, Carlos Javier Rincón Rodríguez

**Affiliations:** 1 Semillero de Bioestadística, Facultad de Medicina, Pontificia Universidad Javeriana, Bogotá, Colombia.; 2 Departamento de Epidemiología Clínica y Bioestadística, Pontificia Universidad Javeriana, Bogotá, Colombia.

**Keywords:** Tamaño de la Muestra, Ensayos Clínicos, Pruebas de Hipótesis

## Abstract

En este artículo se introducen los ensayos clínicos aleatorizados y conceptos básicos de la inferencia estadística. Se presenta como calcular el tamaño de muestra por tipo de desenlace e hipótesis a probar, junto con el código en el lenguaje de programación R para realizar su aplicación. Se presentan cuatro métodos para realizar el ajuste del tamaño de muestra original, cuando se planean análisis interinos. De una manera sencilla y concreta se busca realizar una introducción a estos temas, considerando las expresiones matemáticas que soportan los resultados y su implementación en programas estadísticos disponibles. Con el fin de acercar a los estudiantes de áreas de la salud a la estadística y al uso de programas estadísticos, aspectos poco considerados en su formación.

## INTRODUCCIÓN

El enfoque de la medicina ha cambiado desde una mirada inicial paternalista hasta el reduccionismo pragmático. Este cambio ocurrió por la búsqueda de mejorar la calidad de la atención, disminuir los incentivos económicos individuales y priorizar la importancia de la investigación para mejorar la calidad de la evidencia ^1^. La medicina basada en la evidencia surge como un nuevo paradigma en la década de los 90 como sustento científico para la toma de decisiones clínicas y se fundamenta en una jerarquía de tres afirmaciones: a) los ensayos clínicos aleatorizados (ECA) o revisiones sistemáticas de muchos experimentos suelen aportar mayor evidencia que los estudios observacionales; b) los estudios clínicos analíticos aportan mejor evidencia que la sola racionalidad fisiopatológica; y c) los estudios clínicos analíticos aportan más evidencia que el juicio de expertos ^2^.

Obtener resultados válidos de los ECA depende de la calidad de los datos, la cual debe ser suficiente para abordar la pregunta planteada. Para obtener estos datos de calidad, se debe tener un tamaño de muestra lo suficientemente grande para obtener una estimación precisa del efecto de la intervención. Los errores aleatorios no van a afectar la interpretabilidad de los resultados, siempre y cuando, la muestra sea lo suficientemente grande, sin embargo, un error sistemático puede invalidar un estudio ^3^.

El análisis interino consiste en plantear un(os) punto(s) de observación, donde se realizará una evaluación del comportamiento de la muestra hasta ese punto. Dependiendo de los resultados, el comité podrá determinar la pertinencia de seguir con el estudio o finalizarlo en ese momento ^3^. En este artículo se busca realizar una introducción al cálculo del tamaño de muestra por tipo de desenlace e hipótesis. Además de su ajuste por análisis interino, considerando las fórmulas matemáticas y su implementación en programas estadísticos disponibles como el lenguaje de programación R. El objetivo es acercar al personal de la salud a la estadística y al uso de programas, aspectos poco considerados en su formación. Aunque, ya existen diversas fuentes donde se desarrollan los temas anteriores, no existen muchos documentos donde se integre tanto la teoría como la práctica, incluyendo todos los aspectos mencionados anteriormente en los ECA en paralelo. Contar con artículos de revisión en el idioma español, permite a los jóvenes investigadores y profesionales del área de la salud realizar una primera aproximación a estos temas, sin que se genere un rechazo inicial por su complejidad, en un diseño frecuentemente requerido. Conectar las expresiones matemáticas con su implementación en un programa estadístico busca evitar que, nuevamente jóvenes investigadores ejecuten funciones preestablecidas como por ejemplo *TwoSampleMean.Equality* o *TwoSampleMean.NIS* (incluidas en el paquete «TrialSize» ^4^) sin entender de donde se obtienen los resultado que «arroja» el programa, el efecto de los parámetros en el tamaño de muestra o la necesidad de escoger parámetros con valores coherentes con el tipo de hipótesis que se está evaluando. Lo anterior, busca fomentar la comprensión sobre la ejecución mecánica de tareas solo para cumplir el requerimiento de un comité evaluador.

### Ensayos clínicos aleatorizados

El principio de equipoise corresponde a un estado de incertidumbre frente a los resultados terapéuticos de un tratamiento, donde se justifica la realización de un ECA ^5^. Los ECA con grupo control son estudios prospectivos que comparan los desenlaces de alguna(s) intervención(es) con la mejor alternativa disponible. En estos estudios siempre debe primar la seguridad del paciente, por lo que se les debe explicar los posibles beneficios, daños y las alternativas de tratamiento que existen para su condición. Aunque puede tener limitaciones, se considera la mejor alternativa para evaluar la eficacia o seguridad de una intervención ^6^^,^^7^. Se caracteriza por: a) hay una intervención y se compara con un grupo control que puede ser placebo o el tratamiento habitual, b) hay asignación aleatorizada de las intervenciones en la población para reducir un posible sesgo de confusión al obtener grupos homogéneos y un posible sesgo de selección al evitar prever el grupo al cual se asigna el paciente c) el cegamiento de los grupos del tratamiento se puede realizar tanto para los investigadores, pacientes o analistas y esto minimiza posibles sesgos de información ^6^^,^^7^.

Los ECA se han dividido en cuatro fases. La fase I busca determinar posibles efectos tóxicos, absorción, distribución y metabolismo del fármaco en un grupo de 20-80 personas sanas. La fase II se realiza en población enferma para determinar la seguridad y eficacia del fármaco, a partir de marcadores biológicos y evaluando reacciones adversas. La fase III se realiza cuando hay evidencia sobre la seguridad y eficacia de la intervención y se busca información adicional sobre la seguridad y efectividad del fármaco en un mayor número de participantes. Se compara la intervención con la terapia habitual o placebo en un seguimiento a largo plazo para poder identificar posibles efectos secundarios. En la fase IV, después de que la molécula ha sido aprobada para su comercialización, se compara con otros productos existentes en población general; se realiza farmacovigilancia buscando eventos adversos no identificados en la fase III debido a su baja incidencia o a prolongados periodos de aparición ^3^^,^^6^.

En este artículo nos enfocaremos en los estudios de fase III y IV, donde se requiere un cálculo del tamaño de muestra. Adicionalmente, trabajaremos los ECA en paralelo caracterizados por un seguimiento simultáneo a cada grupo al cual fueron asignados ^3^.

### Estadística inferencial

La estadística inferencial permite estimar el comportamiento de toda la población a partir de los resultados obtenidos en una muestra. Este comportamiento, se resume en medidas como medias, proporciones o varianzas, que de ser obtenidas en toda la población, se denominarían parámetros ^7^. Se tienen dos alternativas: los intervalos de confianza y las pruebas de hipótesis; con resultados consistentes, la primera busca un rango de valores que, con un grado de confianza, contenga el parámetro de interés, mientras que la segunda, evalúa una afirmación sobre el parámetro de interés tomando la decisión de rechazarla o no.

Dado que este documento presenta el cálculo del tamaño de muestra en los ECA en paralelo para evaluar afirmaciones sobre parámetros, describiremos el proceso de las pruebas de hipótesis. Inicialmente se plantean dos hipótesis, la hipótesis nula (H_0_) que es una afirmación sobre el parámetro, y la hipótesis alternativa (H_a_) que es su negación; casi siempre se busca comprobar la hipótesis alternativa ^8^ que se relaciona con la pregunta de investigación; al final se decide rechazar o no la H_0_. Teniendo en cuenta que esta decisión depende de los resultados obtenidos únicamente de una muestra, existe la probabilidad de cometer dos errores, el error tipo I o nivel de significancia (α) que ocurre al rechazar la H_0_ cuando es verdadera y el error tipo II (β), ocurre al no rechazar la H_0_ siendo esta falsa ^9^. Lo opuesto al error tipo I es el nivel de confianza (1-α) y corresponde a la probabilidad de no rechazar la H_0_ cuando esta es verdadera, y el opuesto del error tipo II (1-β), que es el poder, es la probabilidad de rechazar la H_0_ cuando está es falsa ^9^. Al realizar una prueba de hipótesis, se busca que la probabilidad de cometer el error tipo I y II sea baja, lo cual, implica que el nivel de confianza y poder tengan una probabilidad alta (típicamente: α=0,05 y β=0,1 o 0,2). Para poder garantizar estos valores se requiere realizar el cálculo del tamaño de muestra.

Para tomar la decisión de rechazar la H_0_, se realiza una operación a partir de los valores observados en la muestra (estadístico de prueba) y se contrasta con el comportamiento que debería presentar si la H_0_ fuese verdadera. Si el valor observado por el estadístico de prueba es poco probable, esto evidencia que la H_0_ es falsa y se rechaza en favor de la H_a_, de lo contrario se dice que no hay evidencia para rechazar la H_0_. La probabilidad que refleja esta evidencia «a favor» o «en contra» de la H_0_ se denomina valor de p ^10^^,^^11^, y es igual a la probabilidad, asumiendo que la hipótesis nula es verdadera, de obtener un valor del estadístico de prueba «…tan extremo o más (en la dirección adecuada de la H_a_) que el valor calculado en realidad» ^11^^,^^12^; finalmente se rechaza la H_0_ bajo la premisa de un valor de p<α. La significancia estadística, comúnmente evaluada mediante el valor de p, no da cuenta de la significancia clínica; hablamos de significancia estadística cuando se cumple la premisa de un valor de p<α, mientras que la significancia clínica es definida por aquellos resultados que mejoren la funcionalidad física, mental y social del paciente, que puede llegar a ocasionar una mejoría en su calidad o cantidad de vida, dependiendo del contexto ^14^.

### Tipos de hipótesis

En un ECA, se pueden tener diferentes preguntas de investigación que se relacionan con cuatro formas distintas de plantear la H_0_. El cálculo del tamaño de muestra depende del tipo de hipótesis a probar, por lo tanto, en la Tabla 1 se presentan sus definiciones junto con un ejemplo.

**Tabla 1:** Tipos de hipótesis en los ensayos clínicos aleatorizados.

Tipo	Definición ^24^	Hipótesis ^25^^,^^26^	Ejemplo	
			Hipótesis	Interpretación
Igualdad	Evalúa si hay diferencias entre el grupo tratamiento y grupo control.	H_0_: No hay una diferencia entre las dos terapias	La presión sobre la proyección esternal estimada de la válvula aórtica en el esternón no se asocia con un cambio en los parámetros hemodinámicos del paciente hipotenso.	Los pacientes a quienes se les realizó una presión de 6 mm de profundidad sobre la proyección esternal estimada de la válvula aórtica en el esternón, mantenida por 90 s, determina una evolución decreciente de forma homogénea en los parámetros de tensión arterial y frecuencia cardiaca ^27^.
		H_a_: Hay una diferencia entre las dos terapias	La presión sobre la proyección esternal estimada de la válvula aórtica en el esternón se asocia con un cambio en los parámetros hemodinámicos del paciente hipotenso.	
No inferioridad	Evalúa si el efecto de un tratamiento nuevo (cuyo efecto es inferior del tratamiento convencional, pero mayor que el placebo), se encuentra dentro de un rango aceptado y, se establece a partir de la mejor evidencia disponible. Esta diferencia se justifica por efectos secundarios o facilidades para su consecución.	H_0_: El efecto de la nueva intervención es menor o igual al placebo	El nuevo anticomicial presenta la misma efectividad que el placebo.	El nuevo anticomicial, aunque es mejor tolerada que la terapia convencional, es menos efectivo clínica y estadísticamente, por lo que no puede ser recomendado como primera línea ^28^.
		H_a_: El efecto de la nueva intervención es mayor que el placebo	El nuevo anticomicial presenta mayor efectividad que el placebo.	
Superioridad	Busca evaluar si una nueva intervención genera mejores desenlaces clínicos respecto a una terapia bien establecida o placebo	H_0_: La nueva intervención no es superior a la terapia establecida	El voluntariado no reduce el aislamiento social ni impacta en mejores desenlaces en salud mental.	El voluntariado no demostró ser superior comparado con el grupo control respecto a desenlaces en salud mental o aislamiento ^29^.
		H_a_: La nueva intervención es superior a la terapia establecida	El voluntariado reduce el aislamiento social e impacta en mejores desenlaces en salud mental.	
Equivalencia	Busca evaluar si el efecto del tratamiento es idéntico al de otra terapia.	H_0_: Las terapias no son equivalentes	La inclusión en el tratamiento para el síndrome de ovario poliquístico de metformina, asociada con anticonceptivos orales, no es igual de efectivo que la monoterapia con solo anticonceptivos orales.	Se evidenció una mayor efectividad en la terapia combinada dado por una remisión ecográfica más corta, así como menores síntomas, y una tasa de recurrencia a los 3 meses inferior en comparación al grupo de estudio que recibió monoterapia ^30^.
		H_a_: Las terapias son equivalentes.	La administración de monoterapia con anticonceptivos orales es igual de efectivo que la terapia de anticonceptivos orales más metformina para el tratamiento del síndrome de ovario poliquístico.	

H_0_: hipótesis nula, H_a_: hipótesis alternativa

### Tamaño de muestra

Generalmente, no es posible observar a toda la población, por lo tanto, se requiere de una muestra de tamaño específico (*n*) que represente su comportamiento. A medida que el tamaño de muestra va aumentando, los resultados se aproximan al de la población, a tal fin que a partir de un tamaño específico, los resultados no van a presentar grandes cambios haciendo innecesario seguir recolectando a participantes ^15^. Reclutar más sujetos de lo necesario aumenta tanto la complejidad en la operación logística como los costos, y plantea un dilema ético al asignar innecesariamente sujetos a un tratamiento que no ha probado su beneficio. Por otro lado, definir un tamaño de muestra muy pequeño, implica un alto riesgo de que se presenten el error tipo II mencionado anteriormente. El cálculo del tamaño de muestra permite determinar si un estudio es factible basado en supuestos *a priori*, dados por el poder, la significancia y antecedentes de estudios previos que abordan la misma pregunta de investigación, teniendo en cuenta las consideraciones éticas de someter personas a un experimento ^13^^,^^16^.

Adicionalmente, en la realización de un ECA surge la posibilidad de ir observado los resultados obtenidos a medida que se va recolectando la muestra. Lo anterior se denomina «análisis interinos», los cuales deben ser planeados desde el inicio de la investigación en la elaboración del protocolo. Estos análisis adicionales aumentan la posibilidad de que se presenten los errores tipo I y II, por tal motivo, se debe ajustar el tamaño de muestra para mantener un nivel de confianza y poder global en toda la realización del ECA. Lo anterior, refleja la importancia del cálculo del tamaño de muestra, por lo tanto, en este artículo se presenta como realizar el cálculo de tamaño de muestra en los ECA mostrando la expresión de donde se obtiene y su aplicación utilizando el lenguaje de programación R ^17^. Adicionalmente, se presenta como realizar el ajuste por análisis interinos junto con un ejemplo.

## MATERIALES Y MÉTODOS

A partir de la revisión del libro «*Sample size calculations in clinical research*» realizado por Chow *et al.*^13^, en este artículo se presenta como calcular el tamaño de muestra para un ECA en paralelo, por: 1) tipo de desenlace (dicotómico, continuo) y 2) tipo de hipótesis a evaluar (de igualdad, no inferioridad, superioridad y equivalencia). Se incluyen las expresiones matemáticas correspondientes y el código para crear una función en los programas R ^17^ y RStudio ^18^. Para el uso de este código, se requiere que el lector tenga un conocimiento básico sobre el uso de estos programas, en donde debe copiar la función y ejecutarla; ya creada la función se puede utilizar incluyendo los parámetros requeridos que se describen en la sección de resultados. Para cada escenario, se incluye un ejemplo con datos ficticios y se mencionan consideraciones específicas relacionadas con los parámetros de las funciones.

A continuación, se describen los métodos de *Pocock*, *O’Brien y Fleming*, *Wang y Tsiatis* e *Inner Wedge* para realizar el ajuste del tamaño de muestra original, obtenido a partir de las funciones creadas anteriormente, para realizar el análisis interino. El ajuste consiste en multiplicar el tamaño de muestra original por los coeficientes incluidos en los Anexos 1 a 4 dependiendo del método que se utilice, y considerando el número de evaluaciones planeadas (*R*), el poder y nivel de significancia definido para el estudio. Adicionalmente, se incluye la expresión del estadístico de prueba que se utiliza en cada evaluación por tipo de desenlace, con base en la información de los participantes que van ingresando al estudio. En resumen, de cada método se presentan: 1) los valores críticos que corresponden a los valores de la distribución normal estándar que determinan la zona de rechazo para evaluar la hipótesis nula en cada momento, y 2) los coeficientes para el ajuste del cálculo del tamaño de muestra.

### Cálculo de tamaño de muestra desenlace dicotómico

Como ejemplo, asumimos que se van a comparar dos tratamientos y el desenlace de interés es la proporción de fallecidos. Para todas las expresiones que se presentan a continuación, se denota p_T_ y p_C_ como la proporción de fallecidos en el grupo de tratamiento y en el grupo de control, respectivamente; ϵ es la diferencia esperada entre estas dos proporciones (ϵ=p_T_-p_C_ ), δ es el margen de tolerancia o superioridad definido por los investigadores y k es la razón entre el tamaño de muestra del grupo de tratamiento y el grupo control (k=n_T_/n_C_), es decir que n_T_=kn_C_. Finalmente, denotamos α y β como el error tipo I y II, respectivamente; y z_(q)_ como el percentil q de la función de distribución normal estándar. En la Tabla 2, se presentan las expresiones para obtener *n*_*C*_ , y, en el recuadro, el código en el lenguaje de programación R que crea una función para su implementación, junto con un ejemplo donde *α=0,05, β =0,2* y *k=1*.

**Tabla 2:** Tipos de hipótesis con desenlace dicotómico y su código en el lenguaje de programación R.

En las cuatro hipótesis, a menor diferencia esperada (ϵ) y proporciones cercanas a 0,5, mayor es el tamaño de muestra. Al probar una hipótesis de no inferioridad, si a mayor proporción del evento mayor efectividad, entonces *δ<0*; sí a menor proporción del evento mayor efectividad entonces *δ>0*. Al probar una hipótesis de superioridad, si a mayor proporción del evento mayor efectividad entonces *δ>0*; sí a menor proporción del desenlace mayor efectividad entonces *δ<0*. Al probar una hipótesis de equivalencia, siempre *δ>0*.

### Cálculo de tamaño de muestra desenlace continuo

Como ejemplo, asumimos que se van a comparar dos tratamientos donde el desenlace es la presión arterial sistólica en mmHg (PAS). Para todas las expresiones que se presentan a continuación, se denota *μ*_*T*_ y *μ*_*C*_ como la media de la PAS en el grupo de tratamiento y en el grupo de control, respectivamente; ϵ es la diferencia esperada entre las dos medias ϵ*=μ*_*T*_ *-μ*_*C*_ y es la desviación estándar de las dos muestras en conjunto.*δ, k, α, β y z*_*(q)*_ representan los mismos valores que en la sección anterior. En la Tabla 3, se presentan las expresiones para obtener el código en el lenguaje de programación R para su implementación con un ejemplo donde *α=0,05, β=0,2* y *k=1*.

**Tabla 3:** Tipos de hipótesis por desenlace continuo y código en lenguaje de programación R.

En las cuatro hipótesis, a mayor *s* y a menor ϵ se requiere mayor tamaño de muestra. Al probar una hipótesis de no inferioridad, si a mayor *μ* mayor efectividad entonces *δ<0*; sí a menor *μ* mayor efectividad entonces *δ>0*. Al probar una hipótesis de superioridad, si a mayor *μ* mayor efectividad entonces *δ>0*; sí a menor *μ* mayor efectividad entonces *δ<0*. Al probar una hipótesis de equivalencia, siempre *δ>0*.

## RESULTADOS

### Análisis interino

En un ECA se puede ir probando la hipótesis del estudio de manera secuencial a medida que se va recolectando la muestra, dando la posibilidad de suspender la recolección si se identifica tempranamente un claro beneficio de la intervención. Dependiendo el número de evaluaciones *(R)* que se programen, se requiere ajustar el tamaño de muestra inicial para mantener el nivel de significancia global del estudio, y establecer los valores críticos sobre la distribución del estadístico de prueba para rechazar o no la hipótesis nula en cada evaluación. Las *R* evaluaciones se realizan a medida que se van acumulando n/R sujetos, y el estadístico de prueba *z*_*r*_ *(r=1,2,...,R)* para un desenlace dicotómico es igual a:



donde *n*_*r*_ , p̂_T,r_ y p̂_C,r_ son el tamaño de muestra por grupo de intervención y las proporciones estimadas del desenlace al momento de la evaluación *r* del grupo de tratamiento y el grupo control, respectivamente.

Para un desenlace continuo, el estadístico de prueba es igual a:



donde n_r_,  y  son el tamaño de muestra en cada grupo de intervención, y las varianzas estimadas al momento de la r_ésima evaluación del grupo de tratamiento y el grupo control, respectivamente. *x*_*Tj*_ y *x*_*Cj*_ son los valores observados del desenlace en cada sujeto recolectado hasta el momento *r*.

Se presentan cuatro métodos que permiten realizar el ajuste del tamaño de muestra dependiendo del número de evaluaciones programadas, el nivel de significancia y el poder establecido en una hipótesis de igualdad. Primero, el método de *Pocock*, donde el ajuste del tamaño de muestra se realiza multiplicando el tamaño de muestra obtenido inicialmente de las expresiones presentadas en la sección anterior, por los coeficientes incluidos en el Anexo 1, según el número de evaluaciones y los niveles de significancia y poder establecido. Ahora, si *|z*_*r*_ *|>CP*_*(r,α)*_ se rechaza la *H*_*0*_ y se suspende la recolección, en caso contrario, la recolección continua. Los valores críticos *CP*_*(r,α)*_ se presentan en el Anexo 1 para *R y α* definidos. El segundo método es el de *O’Brien y Fleming* y los coeficientes para realizar el ajuste del tamaño de muestra inicial se presentan en el Anexo 2. En esta propuesta se rechaza la *H*_*0*_ en cada evaluación si *|z*_*r*_ *|>COF*_*(r,α)*_ *√(R/r)*, de lo contrario continua. Los valores críticos *COF*_*(r,α)*_ se presenta en el Anexo 2 según el número de evaluaciones y el nivel de significancia. El tercer método es el de *Wang y Tsiatis* que incluye un nuevo parámetro Δ ; en el Anexo 3 se incluyen los coeficientes para el ajuste del tamaño de muestra para *α=0,05*. En este método se rechaza *H*_*0*_ si  en caso contrario, continua. Los valores críticos *CWT*_(r,α,Δ)_ se presentan en el Anexo 3 para α=0,05. Los métodos de *Pocock* y *O’Brien y Fleming* son casos particulares del método de *Wang y Tsiatis* cuando Δ=0,5 y Δ=0, respectivamente, por lo tanto, los valores críticos para estos valores de Δ se obtienen de los Anexos 1 y 2. Finalmente, se presenta el método de *Inner Wedge*; en esta propuesta a diferencia de las tres anteriores, se propone dos valores críticos de tal forma que si *|z*_*r*_ *| ≥b*_*r*_ rechaza la *H*_*0*_ y se suspende la recolección, concluyendo que, se encontró un efecto significativo del tratamiento, si *|z*_*r*_ *| <a*_*r*_ no rechaza la *H*_*0*_ y se suspende la recolección, concluyendo que, no se van a encontrar diferencias entre el tratamiento y el control, de lo contrario, continua la recolección. Los valores críticos *a*_*r*_ y *b*_*r*_ son iguales a:



En el Anexo 4 se presentan los valores de *Cw*1 y *Cw*2 para un α=0,05 y un poder de 0,8 y 0,9, y en las columnas *coef.ajuste* se incluyen los coeficientes, por los cuales se debe multiplicar la muestra original para realizar las *R* evaluaciones.

A manera de ejemplo, considere que se quiere comparar el medicamento A vs. placebo y el desenlace es la proporción de muertes al final del seguimiento. En una hipótesis de igualdad, asumiendo *α=0,05, β=0,1, p*_*T*_ *=0,1 y p*_*C*_ *=0,2* (es decir ϵ*=*0,1), para dos grupos del mismo tamaño, k=1, el tamaño de muestra requerido en cada grupo es de 263 sujetos. Si se plantea realizar R=5 evaluaciones, tenemos que por el método de *Pocock*, el tamaño de muestra ajustado es 263 x 1,207=318 por cada grupo y el valor crítico en cada evaluación es *CP*_0,05,r_=2,413. Por el método de *O’Brien y Fleming*, el tamaño de muestra ajustado es 263 x 1,026=270 para cada grupo y los valores críticos para cada evaluación son *COF*_*(r,0,05)*_ =4,562; 3,226; 2,634; 2,281 y 2,040. Por el método de *Wang* y *Tsiatis,* el tamaño de muestra ajustado para cada grupo, con Δ=0,25, es 263 x 1,066=281 y los valores críticos en cada evaluación son CWT_(r;0,05;0,25)_= 3,194;2,686;2,427;2,259 y 2,136. Finalmente, por el método de *Inner Wedge*, el tamaño de muestra ajustado por cada grupo, con Δ=0,25, es 263 x 1,199=316 y los valores críticos para cada evaluación son *a*_*r*_ =0;0,388; 1,072; 1,613 y 2,073 y *b*_*r*_ =3,1; 2,607; 2,355; 2,192 y 2,073.

## DISCUSIÓN

En este artículo presentamos una aproximación al ajuste del tamaño de muestra por análisis interino en ECA en paralelo, partiendo del cálculo del tamaño de muestra original para su posterior ajuste por uno de los cuatro métodos descritos. El documento está dirigido a estudiantes y jóvenes investigadores, principalmente en el área de la salud, en el que encontrarán un contexto inicial sobre los ECA y una revisión sobre los principales conceptos de la inferencia estadística a partir de las pruebas de hipótesis. Buscamos de una manera sencilla y concreta, realizar una introducción a este tema, integrando los diferentes aspectos como las expresiones matemáticas que soportan los resultados y su implementación en programas estadísticos disponibles. Aunque, existen otros recursos disponibles para el cálculo de tamaño de muestra como páginas de internet ^19^ o paquetes en el lenguaje de programación R ^4^, principalmente en idiomas distintos al español, encontramos que brindar la posibilidad de utilizar programas estadísticos que les permitan aplicar la teoría a los estudiantes, da una mayor compresión de estos temas, a diferencia de seguir una secuencia de pasos de forma mecánica, muchas veces sin entender lo que se genera por los distintos programas o recursos disponibles. Lo anterior, permite acercar a los estudiantes de las áreas de la salud, a la estadística y a el uso de programas estadísticos, aspecto muchas veces considerado poco necesario en su formación.

Este artículo permite al lector, la planeación de un ECA en paralelo definiendo el tamaño de muestra y permitiendo la monitorización de los resultados durante el transcurso del estudio. En este punto, se recomienda continuar con la revisión de métodos adicionales que dan mayor flexibilidad al momento de realizar estas evaluaciones, por ejemplo, permitiendo planear las evaluaciones intermedias en fechas específicas y no cuando se van completando un número fijo de participantes en ambos grupos, el cual es la principal restricción de los cuatro métodos presentados en este artículo. El método propuesto por *Lan* y *DeMets*^20^ y paquetes del lenguaje de programación R como *gsDesign*^21^^)^ serán material de interés para seguir profundizando en estos temas.

Finalmente, la decisión de interrumpir la ejecución de un ECA, ya sea porque se observan grandes beneficios, se presentan potenciales daños o es muy improbable encontrar beneficios (futilidad), debe ser tomada por un grupo independiente al de los investigadores, conformado por expertos en el área clínica bajo estudio, en aspectos metodológicos como epidemiólogos o bioestadísticos y en aspectos éticos ^3^^,^^22^^,^^23^. En esta decisión, se deben considerar todos los aspectos necesarios y no sólo el resultado de la evaluación de una prueba estadística. Realizar la planeación de un análisis interino ajustando el tamaño de muestra desde la elaboración del protocolo, permitirá soportar, en mayor medida, el valor de este criterio en la toma de la decisión.
